# The future of practical skills in undergraduate medical education – an explorative Delphi-Study

**DOI:** 10.3205/zma001061

**Published:** 2016-08-15

**Authors:** Katja Anne Dannenberg, Fabian Stroben, Therese Schröder, Anke Thomas, Wolf E. Hautz

**Affiliations:** 1 Charité – Universitätsmedizin Berlin, Lernzentrum (Skills Lab), Berlin, Germany; 2 Charité – Universitätsmedizin Berlin, Department of Emergency Medicine at Campus Benjamin Franklin, Berlin, Germany; 3 Charité – Universitätsmedizin Berlin, Gynecology and Obstetrics Clinic, Berlin, Germany; 4 Inselspital Bern, University Emergency Center, Bern, Switzerland

**Keywords:** Skills, practical skills, clinical skills, medical training, consensus method, Delphi survey, learning goals, outcomes, competencies, NKLM

## Abstract

**Background:** 64% of young medical professionals in Germany do not feel adequately prepared for the practical requirements of the medical profession. The goal of “outcome-orientated training” is to structure medical curricula based on the skills needed when entering the workforce after completing undergraduate medical education, and thus to bridge the gap between the skills graduates have attained and those necessary for a career in the medical profession. Outcome frameworks (OFs) are used for this purpose. In preparation for developing the National Competence-Based Catalogue of Learning Objectives for Medicine (NKLM) – the German OF – the “Consensus Statement of Practical Skills in Undergraduate Medical Education” (which structures the teaching and acquisition of practical skills in Germany and which strongly influenced the “Clinical-Practical Skills” chapter of the NKLM) was published in 2011.

It is not uncommon for at least a decade to elapse between the definition and implementation of an OF and the students’ graduation, which can further increase the gap between necessary and acquired skills. Thus, the purpose of this paper is to posit theses for future development in healthcare and to apply these theses to a current OF.

**Methodology: **Partially structured interviews with experts were used to generate theses pertaining to general, future development in healthcare. These theses were assessed by physician experts based on the likelihood of implementation by the year 2025. The 288 learning goals of the consensus statement were assessed for their relevance for medical education in the interim.

**Results: **11 theses were generated for the development of medicine, and these theses were assessed and discussed by 738 experts. These theses include the increase in diseases associated with old age, the increasing significance of interprofessional cooperation, and the growing prevalence of telemedicine applications. Of the 288 learning goals of the consensus statement, 231 of the goals were assessed as relevant, and 57 were deemed irrelevant for the short-term future.

**Discussion:** The theses on the future of healthcare, which were generated in this study and which were validated by numerous experts, provide indications of future developments of overall requirements for medical school graduates. For example, when applied to the content of the “Clinical-Practical Skills” NKLM chapter, they largely validate the future relevance of developing practical skills while also providing indications for their further development as applied to the consensus statement.

## Zusammenfassung

**Hintergrund: **64% der Berufsanfänger in Deutschland fühlen sich nicht ausreichend auf die praktischen Anforderungen des Arztberufes vorbereitet. Ziel „ergebnisorientierter Ausbildung“ ist es, medizinische Curricula von den am Ende des Studiums notwendigen Kompetenzen ausgehend zu strukturieren und so die Lücke zwischen nötigen und erworbenen Fähigkeiten der Absolventen zu reduzieren. Dazu dienen insbesondere Lernzielkataloge (outcome frameworks OF). Als Vorarbeit zur Entwicklung des Nationalen Kompetenzbasierten Lernzielkatalogs Medizin (NKLM) – des deutschen OF - wurde 2011 das „Konsensusstatement Praktische Fertigkeiten im Medizinstudium“ publiziert, welches Lehre und Erwerb praktischer Fertigkeiten in Deutschland strukturiert und größtenteils in das Kapitel „Klinisch-Praktische Fertigkeiten“ des NKLM einfloss.

Von der Definition über die Implementation eines OF bis zum Abschluss der Studierenden vergeht oftmals jedoch wenigstens ein Jahrzehnt, was die Lücke zwischen nötigen und erworbenen Kompetenzen wieder vergrößern kann. Ziel dieser Arbeit ist es daher, Thesen zur zukünftigen Entwicklung des Gesundheitswesens zu generieren und auf ein bestehendes OF anzuwenden.

**Methodik: **Mittels halbstrukturierter Experteninterviews wurden Thesen zur allgemeinen, zukünftigen Entwicklung im Gesundheitswesen generiert. Diese Thesen wurden durch ärztliche Experten auf die Wahrscheinlichkeit ihres Eintretens bis zum Jahr 2025 hin bewertet. Die 288 Lernziele des Konsensusstatements, wurden von derselben Expertenkohorte in einer 2-stufigen, explorativen Delphi-Befragung auf ihre mittelfristige Relevanz für die ärztliche Ausbildung hin bewertet.

**Ergebnisse: **Es wurden 11 Thesen zur Entwicklung der Medizin generiert und von 738 Experten bewertet und diskutiert. Die Thesen umfassen die Zunahme altersassoziierter Erkrankungen, die zunehmende Bedeutung von interprofessioneller Zusammenarbeit sowie eine steigende Verbreitung von telemedizinischen Anwendungen. Angewendet auf die 288 Lernziele des Konsensusstatements wurden 231 dieser Ziele als relevant und 57 als irrelevant für die mittelfristige Zukunft bewertet.

**Diskussion: **Die in dieser Studie generierten und durch zahlreiche Experten validierten Thesen zur Zukunft des Gesundheitswesens liefern Anhaltspunkte zur zukünftigen Entwicklung der Anforderungen an Absolventen der Medizin insgesamt. Exemplarisch angewandt auf das Konsensusstatement praktische Fertigkeiten als inhaltgebendes Kapitel „Klinisch-Praktische Fertigkeiten“ des NKLM validieren sie dessen zukünftige Relevanz in großen Teilen, während sie gleichzeitig Hinweise für dessen Weiterentwicklung geben.

## Introduction

On the one hand, the significance of obtaining practical skills during undergraduate medical studies has increased significantly in recent years [1], [2]. On the other hand, 64.7% of young medical professionals in Germany state that they do not feel adequately prepared for the practical requirements of the medical profession [3], a figure which is startlingly high, even compared to international data [4], [5]. Possible causes identified by the graduate survey in Cologne (Stosch C et al., unpublished) and a national survey (partially published in [6]), were both the narrow scope of practical training and the inadequate or lacking integration of this training in curricula and examinations. 

In order to bridge the gap between education and training, medical curricula are increasingly oriented toward national framework curricula, known as “outcome frameworks” (OF) [7], [8], which – generally speaking – describe the skills and knowledge which should be obtained during a training period in a competence-oriented fashion. Various outcome definitions exist internationally [9], [10], [11]. The Tuning Project [12] in Europe is an attempt to synchronize the many national OFs currently in existence. The German “National Competence-Based Catalogue of Learning Objectives for Medicine” (NKLM) was developed by the medical faculty association in cooperation with the Society for Medical Education (GMA) [13] and was initially published in June of 2015 after a six-year period of development [14]. In preparation for developing the NKLM, the “Consensus Statement of Practical Skills in Undergraduate Medical Education” was developed by the committee for practical skills of the GMA in 2011 [15]. This consensus statement “can and should have a formative effect on faculties to adjust their curricula in accordance with guidelines” [15] and strongly influenced the “Clinical-Practical Skills” chapter of the NKLM. The recommendations of the consensus statement have been implemented and validated within at least one faculty department [16]. In addition, the statement serves to assist the simulator network – a merger of the DACH region Skillslabs – to structure its simulator database [17].

There are, however, notable differences in content and structure between different OFs [18], [19], which raises the question of which OF should reasonably be referenced for teaching proficiency. In addition, developing medical curricula is generally a lengthy process: the six stages of the Kern cycle as a widely taught model of curriculum development [20], for example, require a considerable period of time between the initial definition of requirements, implementation, evaluation, and adaptation. Furthermore, an average of 6.4 years [21] elapse between beginning undergraduate medical education and beginning to practice medicine [21]. This contrasts starkly with rapid developments in medicine and the use of new technologies which have become ubiquitous. Consequently, there is a risk that the contents of curricula developed based on current OFs are no longer up-to-date when the medical professionals educated accordingly enter the medical profession.

### 1. Object of the Study

The object of this study is to examine the “Consensus Statement of Practical Skills in Undergraduate Medical Education,” and thus an important part of the NKLM, for medium-term sustainability. The results should, on the one hand, serve to provide details for the further development of the NKLM; and on the other hand, help enhance the future stability of OF and curricula by means of overarching trends in healthcare which must yet be identified. The applied explorative Delphi method, as well as its results, can also serve to further develop local and national curricula.

#### 2. The explorative Delphi method

Originally developed in the 1950s as a technique for exploring technical developments in a military context [22], this method had been continually developed in the intervening decades [23] and is now considered an established method for analyzing uncertain developments and identifying strategic treatment options [22], [24], [25]. In principle, the Delphi method serves to collect group opinions and to focus group communications [24], as well as to qualitatively and quantitatively assess uncertain facts [24]. Although widely varied definitions of the Delphi method exist [24], certain common basic principles can be identified: anonymity of experts, multiple repetitions of the survey, statistical summary of group opinion, and controlled feedback [22]. The use of the Delphi method has been tested in various contexts, though here it is predominantly of interest to sufficiently documented applications in medical education research, such as for developing guidelines [26], [27], [28], [29]. 

## Methods

**Figure 1:**
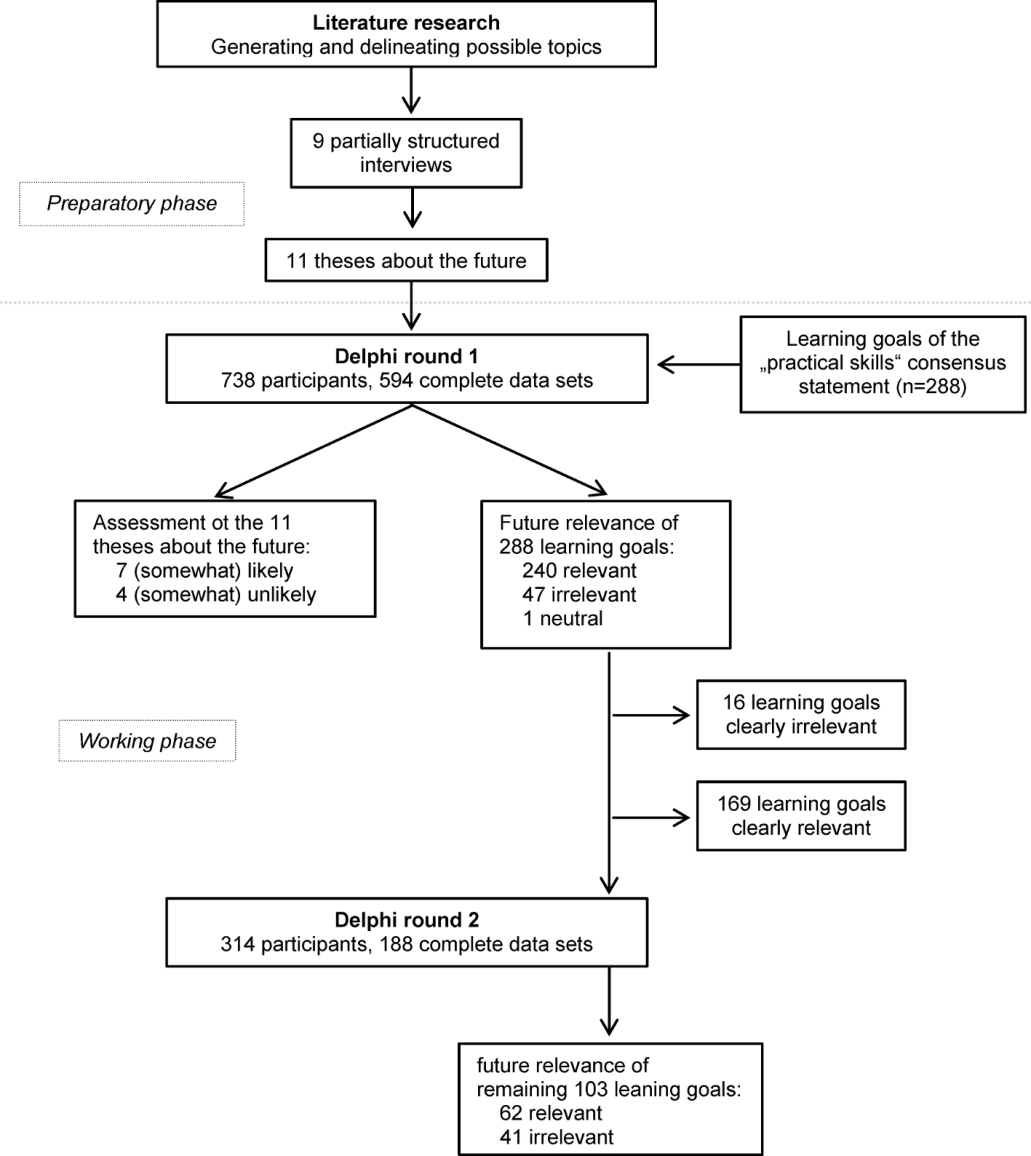
Study design and results overview.

The project was structured into preparatory and working phases. During preparations, literary research followed by partially structured stakeholder interviews was used to develop theses about developments in healthcare. These theses were then assessed by means of an expert survey. In addition, the same expert cohort assessed the 288 learning goals of the “Consensus Statement of Practical Skills in Undergraduate Medical Education” within the framework of a 2-level, explorative Delphi survey. The course of the study is depicted in Figure 1 (Fig. 1).

### 1. Preparatory Phase: Theses on healthcare development

Guidelines for partially structured interview with various healthcare practitioners were developed by means of selective literature research. The topics discussed in the interview included the following:

- The future development of healthcare
- Potential changes to care and to the disease spectrum
- Changes in medical technology and telemedicine
- Interdisciplinarity and cooperation with other occupational groups
- Future changes to undergraduate medical education
- Medical occupations in Germany and abroad
- Medical skills needed in the future

During the preparatory phase, 9 interviews were conducted with experts in the fields of public health, medical technology and pedagogy, clinical and outpatient, practical skills, and students of human medicine (cf. Table 1 (Tab. 1) for details). Interview partners were chosen by means of a “purposive sampling strategy” [30] with the goal of obtaining as broad a spectrum of perspectives on these topics as possible. All interviewed experts were prepared for the interview. Interviews lasted an average of 22 minutes. 

The interviewees’ answers were then grouped by topic using a qualitative text analysis and summarized as theses by means of inductive categorization per Mayring [31] by an interdisciplinary research group comprised of two students of futures studies, including one nurse and one student of human medicine with paramedic training, one practicing physician, and one computer scientist. The goal of the Mayring analysis is to systematically process the written communications at hand, and to identify similarities and differences [32]. The principles of categorization were a) category selectivity, and b) a high level of abstraction of the same. 

#### 2. Working Phase: Expert interviews on future theses generated, and on consensus statement learning goals

After generating theses, their probability of occurrence was assessed within the framework of an expert interview. The individual learning goals of the “Practical Skills in Undergraduate Medical Education” consensus statement were then assessed by the same experts within the framework of a two-level Delphi study. Physicians in all German medical university hospitals whose email addresses were available on the internet, as well as established physicians, were contacted via email to request their participation in the study. In addition to these 8,000 physicians contacted, others were approached at conferences (e.g., the Skills-Lab Symposium 2012) to request their participation in the study.

Each participant then assessed the probability of occurrence of these theses on the future of medical care (generated during the preparatory phase) using a 4-level Likert scale (1 – very likely to 4 – very unlikely). Afterward, each participant was then assigned randomly to a group of ten in order to assess the future relevance of the consensus statement learning goals. This statement defines 288 learning goals assigned to one of 16 organ systems. There is a statement for three different training stages (“clinical traineeship, practical year, advanced training”) based on a three-tiered scale for each learning goal, to what extent this should be mastered (“seen demonstrated, performed under supervision, performed repeatedly”), and the survey further distinguishes between core and elective goals [15]. Each group was asked to assess a portion of the consensus statement learning goals (ca. 30/group) vis-à-vis their relevance for general medical training up to completing undergraduate medical education in the year 2025 using a 4-level Likert scale (1 – highly relevant to 4 – not at all relevant). The learning goal assessments were depicted based on the degree of mastery required by the advanced training stage, as stipulated in the consensus statement.

After round 1 assessments and individual review of the results by the research group, the round 2 learning goals to be assessed were determined by consensus. Selection criteria included a wide distribution of assessment and the estimated significance of each learning goal as assessed in round 1. The learning goals were then re-evaluated by physicians participating in round 2, who were provided with the result of round 1 evaluations. Registered participants were assigned randomly to two groups for this purpose. Each group re-evaluated circa 50 learning goals.

#### 3. Data evaluation

The online interview was conducted using LimeSurvey (http://www.limesurvey.org). Data evaluation was conducted with Microsoft Excel (Microsoft Corporation, Redmond, WA, USA) and IBM SPSS Statistics 21.0 (SPSS Inc., Chicago, IL, USA).

## Results

### 1. Participants

738 experts registered online for round 1 of the study, and 594 complete data sets (19.5% dropout rate) were usable. 314 experts were registered for round 2, and 188 complete data sets (40.1% dropout rate) were usable. Since the learning goals were assessed within the context of the organ system assigned to them, we only took complete data sets into consideration. 

Participants were only asked to assess theses about the future during round 1. Partially-completed questionnaires were taken into consideration here, as the theses about the future can logically be interpreted on an individual basis. For this purpose, 651 expert opinions (11.8% dropout rate) were available.

A large portion of experts in round 1 had more than one year of work experience (96.0%), and 137 experts had been practicing for more than 15 years. The overwhelming majority of physicians worked on an inpatient basis in maximum-care hospitals (87.9%). Study participants represented a total of 26 disciplines. The number of experts in round 2 was smaller, though the characteristics of their working environments were similar. In comparison to German Medical Association (BÄK) statistics from 2014, the proportion of inpatient physicians is large (88.6% in round 1 and 79.3% in round 2, compared to 51.0% by BÄK figures), and the exact distribution of specialties also differed somewhat. Overall, 57.8% (round 1) and 53.7% (round 2) of experts work in disciplines such as surgery, internal medicine, or anesthesia, in addition to general medicine. This is consistent with BÄK data, which lists this figure at 48.8% [http://www.bundesaerztekammer.de/fileadmin/user_upload/downloads/pdf-Ordner/Statistik2014/Stat14AbbTab.pdf]. An exact analysis of the participant cohort in comparison to BÄK figures from 2014 is depicted in Table 2 (Tab. 2). 

#### 2. Theses on the future of healthcare

A total of 11 theses on the future of health care were derived from the partially structured interviews (cf. Table 3 (Tab. 3)). 

**Figure 2:**
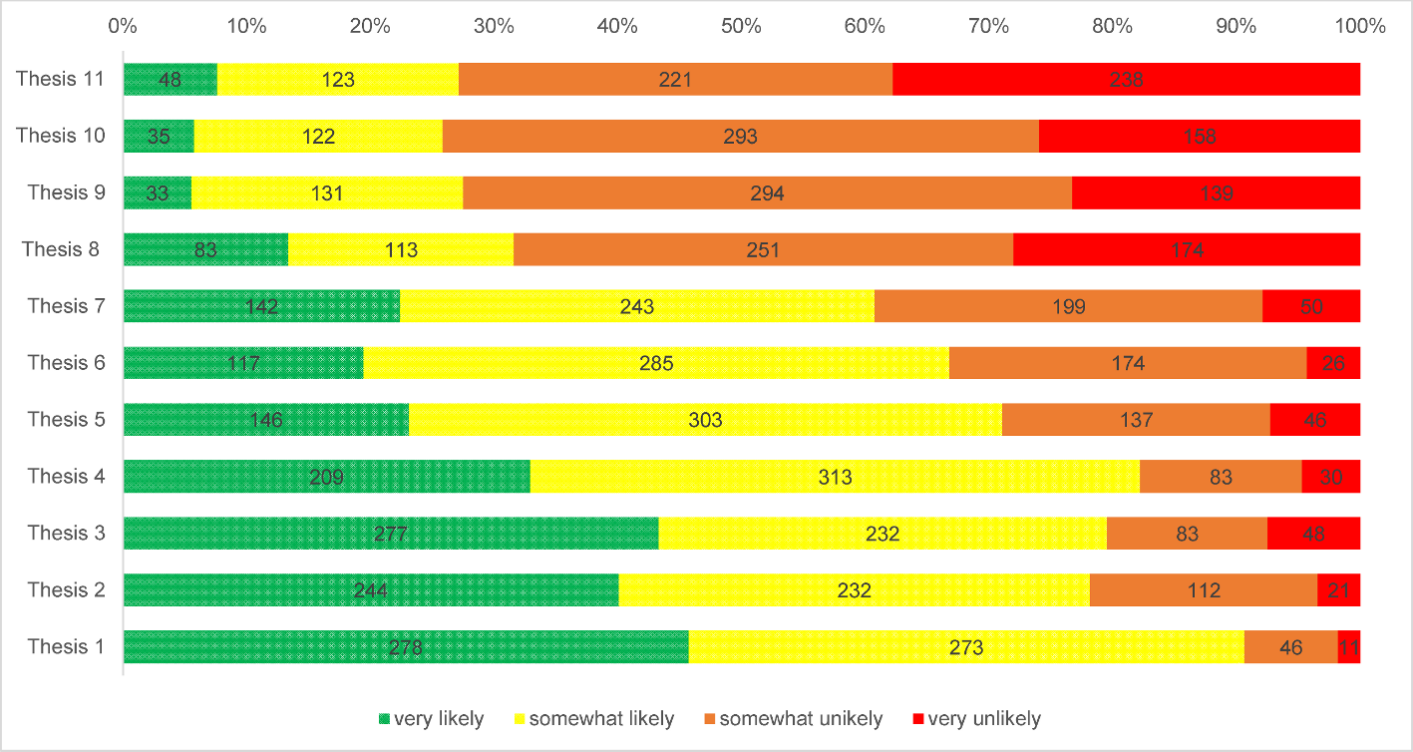
Graphical depiction of the experts’ assessment of the 11 theses on the future of healthcare. See tab. 3 for allocation of these theses.

The theses on the future of healthcare trends generated in the preparatory phase are listed below. It was assumed that these theses were listed in accordance with expert assessment (cf. Table 3 (Tab. 3) and Figure 2 (Fig. 2)): 

Aspects of managing dementia are becoming significantly more important in physician communication. Increasingly balanced patient-physician relationships are emphasizing non-authoritarian forms of discussion and reasoning. 

Increasing mechanization also poses a barrier to entry in the medical field: the relevance of purely manual skills is decreasing, yet IT technologies still cannot determine medical history or diagnoses, requiring the skills of doctors. Diagnostics and patient monitoring will lead to less physical contact, and patients prefer internet and smartphones for this purpose.

The physician remains the personal point of contact in established practice concepts. Duties once performed purely by physicians are, however, increasingly being delegated or substituted. Mobile treatment concepts of primary care are not catching on.

Business economic considerations are slowly moving into focus: while business economic and organizational aspects are included in training, patients’ financial concerns also play a role in the type of care and treatment. 

#### 3. Assessment of learning goals

288 learning goals were assessed by experts in the 2 rounds of the study. The average of all expert assessments was used to determine whether a learning goals was deemed relevant or irrelevant. In round 1 of the Delphi study, 240 learning goals were assessed as relevant or highly relevant (average<2.5) and 57 were assessed as somewhat relevant or irrelevant (average>2.5), while 1 learning goal was assessed as neither (average=2.5).

After reviewing the results of round 1, 103 learning goals were selected for assessment in round 2 based on the distribution of round 1 results; of these, 71 were assessed as relevant, 31 were assessed as irrelevant, and 1 was assessed as neutral in round 1. In round 2, experts assessed 62 learning goals as relevant and 41 as irrelevant. In comparing the two rounds, 13 learning goals (12.6%) were rated less relevant and 4 (3.9%) were rated as more relevant in round 2. A total of 231 learning goals were considered relevant and 57 learning goals were considered irrelevant. Figure 1 (Fig. 1) depicts an overview of these results.

A more extensive review based on organ systems revealed that a large portion of learning goals for the sensory organ system (65.0%) were assessed as irrelevant for the future. Likewise, the future relevance of numerous learning goals associated with the skin, urogenital, and GI tract organs (≥30.0% each) was called into question. 

31 of the 55 (56.4%) elective learning goals were assessed as irrelevant for the future – on the other hand, more than half (54.4%) of non-relevant learning goals are elective. Only 8% of the learning goals and skills which should be mastered upon completing advanced training were assessed as irrelevant for the future, yet 42% of skills were assessed at the lowest level of skill (“seen demonstrated”). The exact results are depicted in Table 4 (Tab. 4). 

The online appendix of this study shows an overview of all consensus statement learning goals and their assessments in the two rounds of the Delphi study. 

## Discussion

This paper attempts, on the one hand, to anticipate future global requirements for medical school graduates; and on the other, to concretely examine the future relevance of practical medical skills as an example of a limited scope of competency in undergraduate medical studies. For the latter, the learning goals of the consensus statement of practical skills in undergraduate medical studies was assessed by means of an explorative Delphi study as preliminary work to the NKLM “clinical-practical skills” chapter.

The panel of experts in the Delphi study possesses many years of work experience and represents nearly all medical specialties. The majority of experts work in maximum-care hospitals, including university hospitals in which undergraduate training is primarily conducted in Germany. Though the experts’ more intensive knowledge has influenced the contents and requirements of the study, outpatient physicians (who are needed primarily for contributing opinions on these theses about the future of medical care) are underrepresented. We can only speculate on the reasons for which so few outpatient physicians participated in the study. 

At the beginning of this paper, 11 theses for relevant topics for future medical education were identified. We deliberately chose not to provide an OF as a basis for the interviews, as national OFs differ substantially in structure [18] as well as content [19]. 

An American group has already published a similar approach [33] which did not, however, take any further validation steps for its theses in comparison to our study. Below, we will discuss a few theses of this study in the context of their assessment by participating experts and derive implications for medical education: 

The current demographic shift has caused an increase in diseases associated with advanced age, such as mild cognitive impairment and dementia [34], [35]. The learning goals catalog lists two learning goals which could be attributed to dementia illnesses. Determining the medical history of elderly patients and performing simple test procedures such as geriatric assessments or falling risk tests were both assessed as relevant for the future. However, emphasizing geriatric test procedures was listed in the catalog as an elective goal, though it should be a core learning goals according to the experts in this study. 

The state of medical care, especially in rural areas with their own specific requirements [36], is in need of improvement [37]. Experience obtained in these places during voluntary training during undergraduate medical studies seemed to have a positive effect on students’ learning and career choices [38] and could improve primary care. The mandatory primary care physician clinical traineeship [39] was recently introduced, and a GMA position paper emphasizes the significance of primary care during undergraduate medical studies [40]. 

In addition to these structural changes, working on an (interprofessional) team and using telemedicine or E-health applications will become more important in the future: delegating work to non-medical personnel increases the effectiveness of primary care [41], [42]. At the same time, learning goals which do not apply solely to physicians, such as applying plaster casts or demonstrating functional taping, have also been assessed as relevant for the future. Beginning to promote interprofessional cooperation during undergraduate medical studies, such as combined courses with trainees or students of other medical care professions, could be one method of implementing the interpersonal aspect of care more intensively during medical education.

In addition, electronic support system (health information technology) resources that are currently available could be used more effectively [42], [43]. Some positive effects of this technology, such as increased activity for COPD patients [44] or improved control of chronic asthma symptoms [45], have already been demonstrated. Integrating this growing field into training and education seems crucial, and could take place by means of telemedicine modules [46]. A dedicated learning goals catalog for e-health and telemedicine has already been published and can be consulted for future developments [47].

In consideration of this knowledge and the high relevance of soft skills and communication ability in this Delphi study, telephone- or internet-based physician-patient interaction could also grow more significant [48]. In order to do this field justice, training for communication skills (e.g., via telephone) should be intensified [49], as has been implemented in individual cases [50], [51]. Older patients in Germany see telemedicine methods more critically, however, and miss personal contact with and direct feedback from their physician [52].

Prioritization of core and elective learning goals in the OF original publication [15] (which were, in part, determined by means of the Delphi methodology and our results) mutually validate each other. More than 90% of the consensus statement learning goals assessed as needing to be mastered and nearly all border area learning goals were assessed by our participants as especially relevant for the future of the medical profession. On the other hand, more than 50% of skills listed in the consensus statement as elective were also assessed as less relevant by our study.

The experts assessed practical learning goals overwhelmingly as relevant for the future, primarily in the large categories of communication skills, soft skills, interinstitutional skills, cardiovascular, and emergency, but also in narrower disciplines such as mental health and the endocrine system. The portion of learning goals not relevant for the future is largest for the sensory organs and for the urogenital and GI tract systems. This could be related to the choice of experts, but could also be due to the fact that the learning goals were phrased in a very specific manner, and thus there are a great many of them. In other catalog categories, more learning goals tended to be summarized as one, which made it difficult for experts to provide a differentiated assessment. In addition, it cannot be determined whether rejection of a learning goal was due to a general lack of future relevance, or because experts considered the learning goal relevant for the future, but believed that the learning goal should be a part of specialty training rather than general medical training.

In a further step, the detailed results of this study (cf. online appendix) could be used, just like other validation studies [16], to re-assess the individual learning goals of the consensus statement and the NKLM, which would contribute to a review of the consensus statement and the NKLM.

Preparing future physicians to practice medicine should be done, in parallel, on as many levels as possible. Numerous OFs, unlike this study, currently name the “self-directed learning” method resulting from “self-assessment” [53] as a significant method of improving the results of undergraduate medical studies [54]. At the same time, there are significant doubts as to the accuracy of self-assessments [53], [55], [56]. Thus, “lifelong learning” based on self-assessment cannot be the only methods of anticipating and addressing future developments.

This should be done at the level of the OF. In addition to instructions on effective, self-directed, and lifelong learning, optimizing current OFs could make significant contributions. Anticipating future developments in conjunction with current research results could, on the one hand, provide important incentives for new content, and on the other hand, explorative Delphi studies could examine current learning goals and OFs with respect to their sustainability and possibly identify deficiencies. In concrete terms, the results of the Delphi study could serve to justify specific revisions to and implementation of the NKLM in different departments. Broad trend-setting decisions on possible future trends in undergraduate medical education can be derived from the assessment of these theses on the future. 

### 1. Limitations

Cognitive bias must be accepted as a significant limitation of any expert survey. This is of particular importance for the application of the explorative Delphi method for assessing issues that are uncertain, per se, as they take place in the future, since the line between rational assessment and the experts’ personal desires or fears could be blurred [57]. The structure of the survey could also have influenced expert opinions. After first assessing the likelihood of implementing theses in the future, experts were then asked to assess the future relevance of learning goals. This could have led to a bias. Though experts were asked to base their assessments on general education leading up to the medical exam, it cannot be determined whether this was actually done, and to what extent experts assessed general education as opposed to specialty training.

The study population of this paper is comprised primarily of inpatient physicians at maximum-care facilities. Consequently, a bias against outpatient treatment methods cannot be ruled out. In addition, colleagues practicing general medicine were underrepresented (just 2.9% of experts), while anesthesiology and intensive care – two highly specialized subjects – were overrepresented, which could explain the strong emphasis on technological trends in the theses generated. One possible explanation for the large number of anesthesiology practitioners represented in the study could be their disproportionate involvement in conveying practical skills. A follow-up survey with primarily outpatient physician seems reasonable.

## Conclusions

The explorative Delphi method provides an adequate opportunity to allow a current outcome framework to be assessed by experts on the basis of its future relevance. In addition, future trends can be anticipated by means of generating and assessing theses. It is important to continually review and adapt current OF and curricula to future developments in order to provide optimal preparation for medical studies graduates for their future daily professional lives.

## Acknowledgements

We would like to thank Sascha Dannenberg for his help and support with technical questions about futures studies, and for his assistance in carrying out the study. In addition, we would like to thank Nicole Ambacher and Daniel Knapp for their support in data collection. Finally, we would like to sincerely thank all of the experts who took part in this study. 

## Data

Data for this article are available from the Dryad Digital Repository: http://dx.doi.org/10.5061/dryad.q4sc8 [58]

## Competing interests

The authors declare that they have no competing interests.

## Supplementary Material

Online attachment – only in german

## Figures and Tables

**Table 1:**
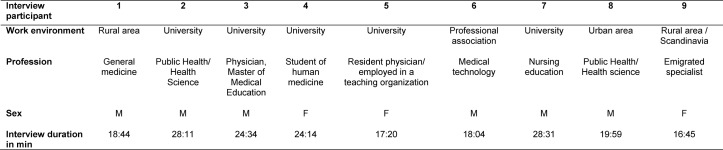
Overview of interview partners

**Table 2:**
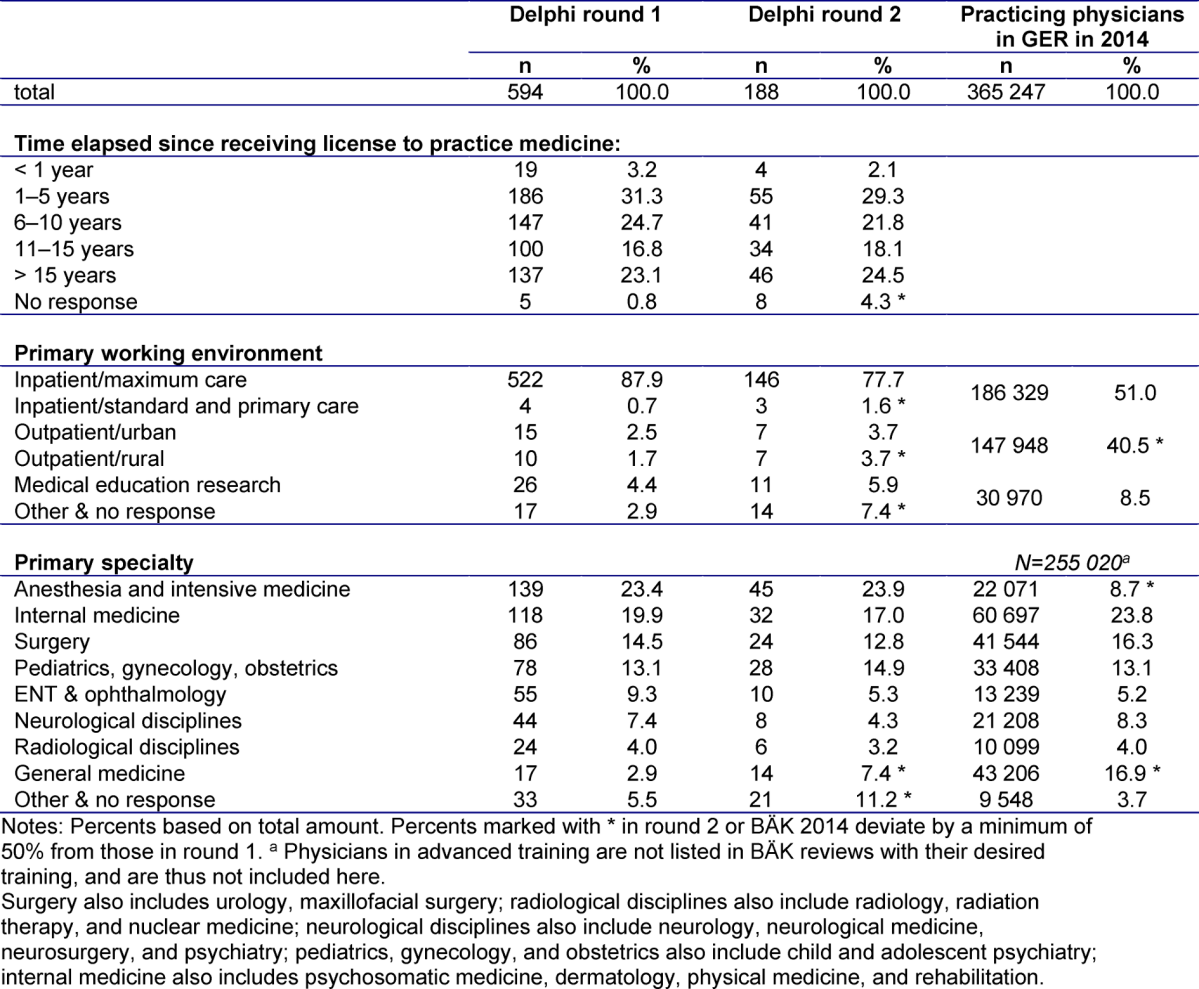
Work experience, environment, and specialties for participants in rounds 1 and 2.

**Table 3:**
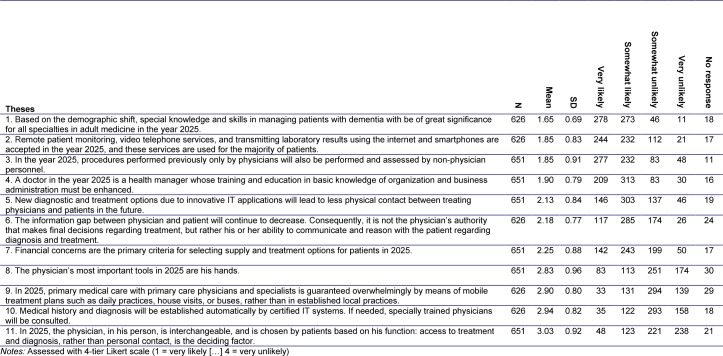
Expert responses (round 1) to the 11 stakeholder theses

**Table 4:**
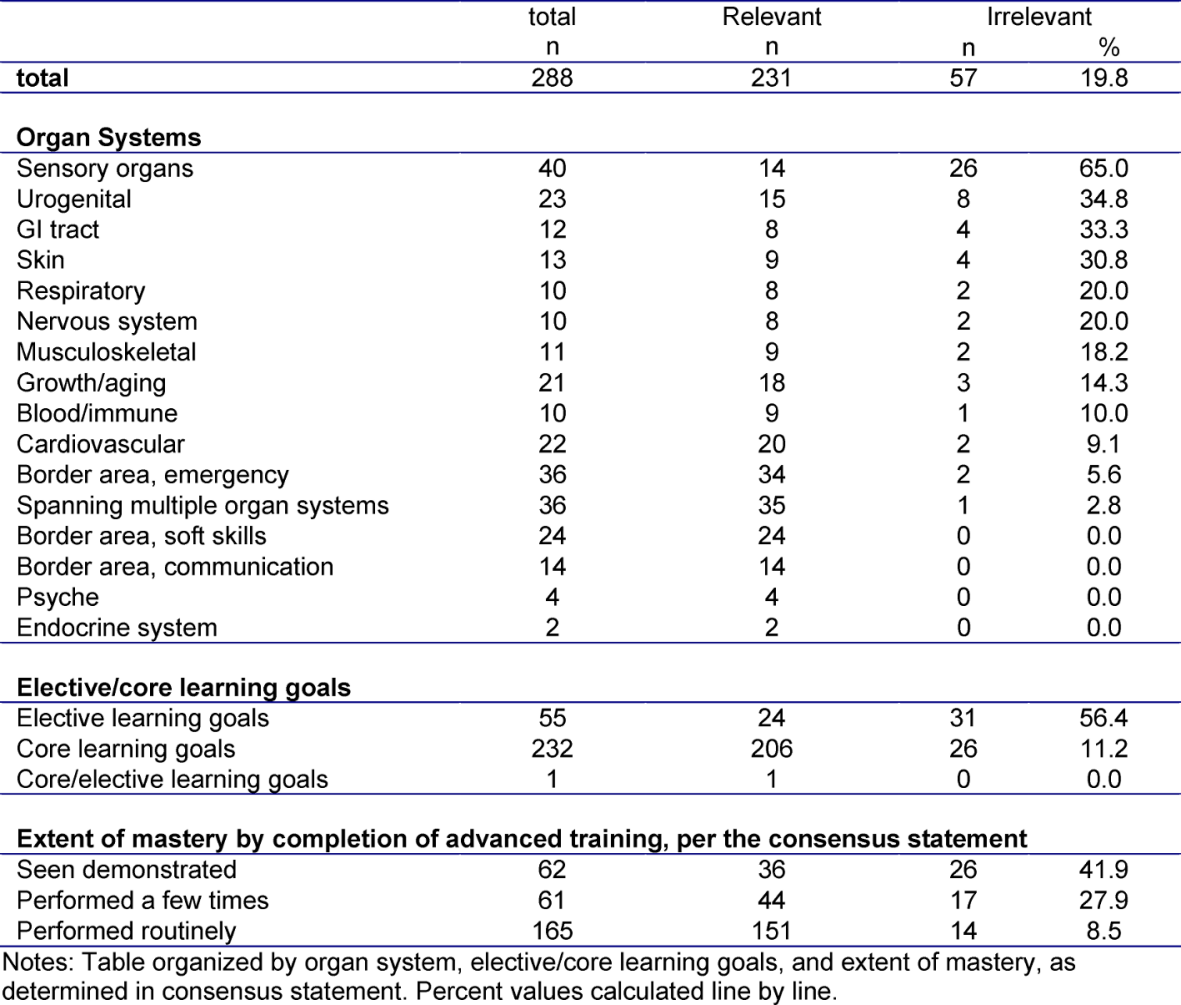
Results of Delphi Rounds 1 + 2
