# Supplementary material for: The future of practical skills in undergraduate medical education – an explorative Delphi-Study
Source: GMS J Med Educ. 2016 Aug 15;33(4):Doc62. doi: 10.3205/zma001061 (PMC5003134; doi:10.3205/zma001061)
Supplement: Online attachment – only in german [file JME-33-62-s-001.pdf]

Nummer Konsensusstatement*	Organsystem	Fertigkeit	Kern-/Wahllernziel	Tiefendimension WB-Reife	Runde 1: Expertengruppe	n	Mittelwert	Standardabweichung	Runde 2: Expertengruppe	n	Mittelwert	Standardabweichung	Abschlussbewertung
1	Atmung	Klinische Untersuchung der Lunge (Inspektion, [Atemfrequenz/-muster], Palpation [Bronchophonie, Stimmfremitus], Perkussion [Lungengrenzen/-Verschieblichkeit], Auskultation)	K	3	1	84	1,12	0,36					zukunftsrelevant
2	Atmung	Bedienung eines einfachen Beatmungsgerätes	K	3	1	84	1,83	0,77					zukunftsrelevant
3	Atmung	Durchführung einer Bronchoskopie	W	1	1	84	2,24	0,63	11	96	2,45	0,72	zukunftsrelevant
4	Atmung	Durchführung einer Koniotomie am Modell	K	1	1	84	2,25	0,89	11	97	2,32	0,77	zukunftsrelevant
5	Atmung	Durchführung einer Lungenfunktionsanalyse (Spirometrie, Bodyplethysmographie)	K	2	1	84	2,14	0,70	11	97	2,36	0,77	zukunftsrelevant
6	Atmung	Durchführung einer Pleurapunktion	K	2	1	84	2,02	0,71					zukunftsrelevant
7	Atmung	Anlage einer Pleuradrainage	W	2	1	84	2,31	0,80	11	99	2,60	0,81	nicht zukunftsrelevant
8	Atmung	Durchführung einer Rhinomanometrie	W	2	1	84	3,34	0,61					nicht zukunftsrelevant
9	Atmung	Durchführung einer Tracheotomie	KW	1	1	84	2,39	0,81	11	101	2,43	0,75	zukunftsrelevant
10	Atmung	Untersuchung des Thorax auf eine Rippenserienfraktur	K	3	1	84	1,92	0,71					zukunftsrelevant
11	Bewegungsapparat	Aktive und passive Untersuchung der oberen Extremität inkl. Schulter, Ellenbogen und Handgelenken sowie der Langfinger und Daumen (insbesondere Inspektion, Auffinden anatomischer Landmarken, Durchführung der Neutral-Null-Methode und der Funktionstests für die Gelenke)	K	3	1	84	1,49	0,59					zukunftsrelevant
12	Bewegungsapparat	Aktive und passive Untersuchung der unteren Extremität inkl. Hüfte, Knie- und Sprunggelenken sowie des Fußes (insbesondere Inspektion, Auffinden anatomischer Landmarken, Durchführung der Neutral-Null-Methode und der Funktionstests für die Gelenke)	K	3	1	84	1,52	0,61					zukunftsrelevant
13	Bewegungsapparat	Aktive und passive Untersuchung der Wirbelsäule (inkl. Inspektion, Neutral-Null-Methode und Funktionstests)	K	3	1	84	1,51	0,59					zukunftsrelevant
14	Bewegungsapparat	Anlegen von Tapeverbänden, funktionelles Taping	K	1	1	84	2,22	0,81	12	96	2,25	0,80	zukunftsrelevant
15	Bewegungsapparat	Durchführung von physikalischer Therapie	W	1	1	84	2,20	0,79	12	94	2,14	0,77	zukunftsrelevant
16	Bewegungsapparat	Durchführung von Physiotherapie	K	1	1	84	2,29	0,80	12	93	2,06	0,69	zukunftsrelevant

*Konsensusstatement "Praktische Fertigkeiten im Medizinstudium" [...]. Schnabel KP et al, GMS Z Med Ausbild, 2011

Nummer Konsensusstatement*	Organsystem	Fertigkeit	Kern-/Wahllernziel	Tiefendimension WB-Reife	Runde 1: Expertengruppe	n	Mittelwert	Standardabweichung	Runde 2: Expertengruppe	n	Mittelwert	Standardabweichung	Abschlussbewertung
17	Bewegungsapparat	Klinische Erhebung der Muskelfunktion	K	3	1	84	1,78	0,75					zukunftsrelevant
18	Bewegungsapparat	Durchführung einer Gelenkpunktion	W	1	1	84	2,28	0,72	12	94	2,54	0,77	nicht zukunftsrelevant
19	Bewegungsapparat	Durchführung von Gips- und Immobilisationstechniken	K	2	1	84	2,06	0,73					zukunftsrelevant
20	Bewegungsapparat	Orthopädietechnik und Orthopädieschuhtechnik	W	1	1	84	2,71	0,74	12	92	2,69	0,67	nicht zukunftsrelevant
21	Bewegungsapparat	Reposition von Luxationen	K	1	1	84	1,84	0,59					zukunftsrelevant
22	Blut Abwehr	Patienten vollständig auf Blutungszeichen untersuchen	K	3	1	83	1,48	0,61					zukunftsrelevant
23	Blut Abwehr	Anfertigung und Mikroskopie eines Blutausstriches	K	3	1	83	2,63	0,78	12	94	2,60	0,91	nicht zukunftsrelevant
24	Blut Abwehr	Bedside Test durchführen können	K	3	1	83	1,46	0,72					zukunftsrelevant
25	Blut Abwehr	Durchführung und Monitoring prophylaktischer und therapeutischer Antikoagulation	K	3	1	83	1,63	0,71					zukunftsrelevant
26	Blut Abwehr	Lymphknotenstatus, einzelne Lymphknoten gezielt auf ihre Beschaffenheit untersuchen (z.B. Konsistenz, Verschieblichkeit, Schmerzhaftigkeit, Überwärmung)	K	3	1	83	1,31	0,52					zukunftsrelevant
27	Blut Abwehr	Kapillare Blutentnahme	K	3	1	83	1,70	0,84					zukunftsrelevant
28	Blut Abwehr	Knochenmarkpunktion/ Knochenstanze	W	1	1	83	2,35	0,63	12	92	2,28	0,74	zukunftsrelevant
29	Blut Abwehr	Lichtmikroskopie (Handhabung)	K	3	1	83	2,39	0,95	12	92	2,19	0,90	zukunftsrelevant
30	Blut Abwehr	Überwachung transfundierter Patienten und klinische Beurteilung von Transfusionsreaktionen	K	3	1	83	1,71	0,79					zukunftsrelevant
31	Blut Abwehr	Verschiedene mikrobiologische Verfahren zum Erregernachweis nach Indikation auswählen und einsetzen können (z.B. Asservation der Probe für Blutkultur, Abstrich, Urinkultur, Nachweisverfahren, Präanalytik)	K	3	1	83	1,84	0,88					zukunftsrelevant
32	Endokrines System	Inspektion und Palpation der Speicheldrüsen	K	1	4	53	2,21	0,84	11	96	2,36	1,00	zukunftsrelevant
33	Endokrines System	Inspektion, Palpation und Auskultation der Schilddrüse	K	3	4	53	1,92	0,76					zukunftsrelevant
34	GI-Trakt	2. Assistenz bei einem visceralchirurgischen Eingriff	K	3	2	47	2,91	0,72	11	101	2,77	0,97	nicht zukunftsrelevant
35	GI-Trakt	24h-pH-Metrie durchführen und beurteilen können	W	1	2	47	3,18	0,70					nicht zukunftsrelevant

*Konsensusstatement "Praktische Fertigkeiten im Medizinstudium" [...]. Schnabel KP et al, GMS Z Med Ausbild, 2011

Nummer Konsensusstatement*	Organsystem	Fertigkeit	Kern-/Wahllernziel	Tiefendimension WB-Reife	Runde 1: Expertengruppe	n	Mittelwert	Standardabweichung	Runde 2: Expertengruppe	n	Mittelwert	Standardabweichung	Abschlussbewertung
36	GI-Trakt	Aszitespunktion	K	1	2	47	1,94	0,79					zukunftsrelevant
37	GI-Trakt	Anamnese zur Beurteilung abdomineller Schmerzen	K	3	2	47	1,19	0,40					zukunftsrelevant
38	GI-Trakt	Klinische Untersuchung des Abdomens durchführen (Inspektion, Palpation [z.B. Abwehrspannung, Druckschmerz, Leberrand, Courvouisierzeichen, Milzvergrößerung, Tumoren, Appendizitis], Perkussion [z.B. Leberdämpfung, Aszites], Auskultation [z.B. Darmgeräusche)	K	3	2	47	1,19	0,40					zukunftsrelevant
39	GI-Trakt	Untersuchung der Leiste (Hernien, Lymphknoten)	K	3	2	47	1,36	0,53					zukunftsrelevant
40	GI-Trakt	Endoskopische Verfahren (Ösophago-Gastro-Duodenoskopie, Coloskopie)	W	1	2	47	1,96	0,69					zukunftsrelevant
41	GI-Trakt	Laktose-Toleranztest durchführen und beurteilen können	W	1	2	47	2,93	0,82					nicht zukunftsrelevant
42	GI-Trakt	Magendarm-Passage anordnen (Indikation stellen, Vorbereitung, Interpretation, Demonstration)	W	1	2	47	2,15	0,93	11	99	2,54	1,02	nicht zukunftsrelevant
43	GI-Trakt	Magensonde legen	K	3	2	47	2,09	0,95	11	97	1,84	1,03	zukunftsrelevant
44	GI-Trakt	MRT- und CT-Untersuchungen des Abdomens interpretieren können	W	2	2	47	2,21	0,78					zukunftsrelevant
45	GI-Trakt	Rektale Untersuchung (auch am Modell)	K	3	2	47	1,66	0,64					zukunftsrelevant
46	Grenzbereich Kommunikation	Eine symtomorientierte, Organsystem-orientierte und allgemeine Anamnese systematisch erheben	K	3	8	60	1,37	0,55					zukunftsrelevant
47	Grenzbereich Kommunikation	Auf besondere Anamnesesituationen eingehen können (ältere Menschen, Kinder, Sprachschwierigkeiten, Behinderungen, Fremdanamnese, Notfallsituation)	K	3	8	60	1,66	0,57					zukunftsrelevant
48	Grenzbereich Kommunikation	Befunde und Diagnosen mitteilen können	K	3	8	60	1,57	0,62					zukunftsrelevant
49	Grenzbereich Kommunikation	Allgemeine Aufklärungsgespräche führen können	K	3	8	60	1,62	0,52					zukunftsrelevant
50	Grenzbereich Kommunikation	Die Einwilligungsfähigkeit von Patienten ermitteln können	K	3	8	60	1,75	0,63					zukunftsrelevant
51	Grenzbereich Kommunikation	Die Einwilligung von Patienten einholen können	K	3	8	60	1,63	0,61					zukunftsrelevant

*Konsensusstatement "Praktische Fertigkeiten im Medizinstudium" [...]. Schnabel KP et al, GMS Z Med Ausbild, 2011

Nummer Konsensusstatement*	Organsystem	Fertigkeit	Kern-/Wahllernziel	Tiefendimension WB-Reife	Runde 1: Expertengruppe	n	Mittelwert	Standardabweichung	Runde 2: Expertengruppe	n	Mittelwert	Standardabweichung	Abschlussbewertung
52	Grenzbereich Kommunikation	Vorgehen bei Untersuchung am Patienten vermitteln können	K	3	8	60	1,57	0,56					zukunftsrelevant
53	Grenzbereich Kommunikation	Schlechte Nachrichten überbringen können (evt. am SP)	K	3	8	60	1,65	0,71					zukunftsrelevant
54	Grenzbereich Kommunikation	Erhobene Befunde dokumentieren können	K	3	8	60	1,64	0,60					zukunftsrelevant
55	Grenzbereich Kommunikation	Mit Patienten am Lebensende/ Sterbenden kommunizieren können	K	3	8	60	1,55	0,62					zukunftsrelevant
56	Grenzbereich Kommunikation	Mit unmotivierten/ nicht zurechnungsfähigen Patienten umgehen können	K	3	8	60	1,93	0,76					zukunftsrelevant
57	Grenzbereich Kommunikation	Sprach- und Sprechstörungen erkennen und adäquat kommunizieren können	K	2	8	60	2,29	0,74	11	101	2,28	0,88	zukunftsrelevant
58	Grenzbereich Kommunikation	Identifikation des Patienten	K	3	8	60	1,98	0,68					zukunftsrelevant
59	Grenzbereich Kommunikation	Zusammenarbeit mit dem Casemanagement, Sozialdienst und Pflegeexperten (z.B. Wundmanagement, Schmerzmanagement)	K	3	8	60	1,92	0,72					zukunftsrelevant
60	Grenzbereich Notfall	Anlegen und bedienen eines Automatischen Externen Defibrillators	K	3	8	59	1,85	0,76					zukunftsrelevant
61	Grenzbereich Notfall	Advanced Life Support (ALS)	K	1	8	59	1,57	0,72					zukunftsrelevant
62	Grenzbereich Notfall	Notfalldokumentation am Beispiel eines Notarztprotokolls	K	2	8	59	2,24	0,75	11	99	1,98	0,72	zukunftsrelevant
63	Grenzbereich Notfall	Bedienen von Spritzenpumpen	K	2	8	59	2,33	0,73	11	97	1,88	0,79	zukunftsrelevant
64	Grenzbereich Notfall	Unterstützende Maßnahmen bei Spontangeburten	K	1	8	59	2,29	0,83	11	97	2,01	0,66	zukunftsrelevant
65	Grenzbereich Notfall	Basic Life Support (BLS) Erwachsene	K	3	8	59	1,46	0,60					zukunftsrelevant
66	Grenzbereich Notfall	Basic Life Support (BLS) Kinder	K	3	8	59	1,68	0,68					zukunftsrelevant
67	Grenzbereich Notfall	Bodycheck im Rahmen eines standardisierten Trauma-Algorithmus	K	2	8	59	1,86	0,71					zukunftsrelevant
68	Grenzbereich Notfall	Sichere Defibrillation/ Kardioversion durchführen können	K	2	8	59	1,83	0,75					zukunftsrelevant
69	Grenzbereich Notfall	Grundprinzipien der Reposition	K	1	8	59	2,39	0,67	11	96	2,57	0,75	nicht zukunftsrelevant

*Konsensusstatement "Praktische Fertigkeiten im Medizinstudium" [...]. Schnabel KP et al, GMS Z Med Ausbild, 2011

Nummer Konsensusstatement*	Organsystem	Fertigkeit	Kern-/Wahllernziel	Tiefendimension WB-Reife	Runde 1: Expertengruppe	n	Mittelwert	Standardabweichung	Runde 2: Expertengruppe	n	Mittelwert	Standardabweichung	Abschlussbewertung
70	Grenzbereich Notfall	Lagerung und Ruhigstellung von verletzen Extremitäten	K	3	9	67	1,94	0,60					zukunftsrelevant
71	Grenzbereich Notfall	Anwendung von Transporthilfsmitteln von Notfallpatienten (Schaufeltrage, Vakuummatratze etc.)	K	2	9	67	2,22	0,71	11	101	2,01	0,69	zukunftsrelevant
72	Grenzbereich Notfall	Überwachung eines Notfallpatienten beim Transport	K	3	9	67	1,94	0,62					zukunftsrelevant
73	Grenzbereich Notfall	Übergabe eines Notfallpatienten	K	3	9	67	1,69	0,60					zukunftsrelevant
74	Grenzbereich Notfall	Hilfestellung bei der Einnahme von Medikamenten geben können	K	3	9	67	2,44	0,86	11	99	1,91	0,89	zukunftsrelevant
75	Grenzbereich Notfall	Intraossäre Punktion	K	2	9	67	2,71	0,85	11	97	2,62	0,95	nicht zukunftsrelevant
76	Grenzbereich Notfall	Katheteranlage (DK, Magen)	K	3	9	67	2,26	0,84	11	97	1,83	0,64	zukunftsrelevant
77	Grenzbereich Notfall	Klinische Diagnosesicherung einer Fraktur	K	2	9	67	1,82	0,60					zukunftsrelevant
78	Grenzbereich Notfall	Lagerungsmaßnahmen des Notfallpatienten (Schocklage etc.)	K	3	9	67	1,50	0,63					zukunftsrelevant
79	Grenzbereich Notfall	Beutel-Maskenbeatmung durchführen	K	2	9	67	1,44	0,63					zukunftsrelevant
80	Grenzbereich Notfall	Anwendung einer supraglottischen Atemhilfe	K	3	9	66	1,98	0,71					zukunftsrelevant
81	Grenzbereich Notfall	Anwendung einer Pocket Maske	K	3	9	66	1,93	0,70					zukunftsrelevant
82	Grenzbereich Notfall	Endotracheale Intubation	K	1	9	66	1,78	0,69					zukunftsrelevant
83	Grenzbereich Notfall	Pupillenreaktion testen	K	2	9	66	1,35	0,51					zukunftsrelevant
84	Grenzbereich Notfall	Umgang mit Blutprodukten (Bedside-Test, Patientenidentifikation, Applikation)	K	3	9	66	1,52	0,68					zukunftsrelevant
85	Grenzbereich Notfall	Sterile Wundabdeckung	K	3	9	66	1,56	0,58					zukunftsrelevant
86	Grenzbereich Notfall	Erstversorgung von Verbrennungen und Verbrühungen	K	1	8	59	2,15	0,71	12	96	1,58	0,72	zukunftsrelevant
87	Grenzbereich Notfall	Primäre Blutstillung	K	2	9	67	1,83	0,73					zukunftsrelevant
88	Grenzbereich Notfall	Blutstillung vital bedrohlicher Blutungen (Kompressionsverband)	K	3	9	67	1,65	0,75					zukunftsrelevant
89	Grenzbereich Notfall	Blutstillung vital bedrohlicher Blutungen (Tourniquet)	K	3	9	66	1,81	0,72					zukunftsrelevant
90	Grenzbereich Notfall	Zugänge anlegen	K	3	9	66	1,32	0,56					zukunftsrelevant
91	Grenzbereich Notfall	HWS Immobilisation mit Cervical-Orthese	K	3	9	66	2,22	0,73	12	93	1,95	0,89	zukunftsrelevant
92	Grenzbereich Notfall	Manuelle HWS Immobilisation	K	3	9	66	1,88	0,64					zukunftsrelevant
93	Grenzbereich Notfall	Pneumothoraxentlastung	K	2	9	66	2,12	0,75	12	96	2,13	0,97	zukunftsrelevant
94	Grenzbereich Notfall	Helmabnahme	K	3	9	66	1,89	0,75					zukunftsrelevant
95	Grenzbereich Notfall	Thoraxdrainage	K	1	9	66	2,11	0,70	11	96	2,09	0,68	zukunftsrelevant

*Konsensusstatement "Praktische Fertigkeiten im Medizinstudium" [...]. Schnabel KP et al, GMS Z Med Ausbild, 2011

Nummer Konsensusstatement*	Organsystem	Fertigkeit	Kern-/Wahllernziel	Tiefendimension WB-Reife	Runde 1: Expertengruppe	n	Mittelwert	Standardabweichung	Runde 2: Expertengruppe	n	Mittelwert	Standardabweichung	Abschlussbewertung
96	Grenzbereich Soft Skills	Anwendung von computergestützten (internetbasierten) Aus- und Fortbildungsmöglichkeiten	K	3	10	73	1,53	0,60					zukunftsrelevant
97	Grenzbereich Soft Skills	Arbeitsweise, Verhaltensregeln und Assistenz im OP	K	3	10	73	1,97	0,73					zukunftsrelevant
98	Grenzbereich Soft Skills	Ausstellen von Dokumenten (z.B. Arbeitsunfähigkeits-bescheinigung, Ein-/Überweisung, Rezept [auch BTM])	K	2	10	73	2,32	0,74	11	101	1,77	0,86	zukunftsrelevant
99	Grenzbereich Soft Skills	Berücksichtigung des Arbeits- und Gesundheitsschutzes in der eigenen ärztlichen Tätigkeit und Verantwortung (z.B. Nadelstichverletzung, rückenschonendes Arbeiten, Recapping, Handschuhtragen, Bereich Hygiene, Arbeitsmedizin...)	K	3	10	73	2,14	0,73	11	99	1,76	0,78	zukunftsrelevant
100	Grenzbereich Soft Skills	Datenschutz	K	3	10	73	1,89	0,70					zukunftsrelevant
101	Grenzbereich Soft Skills	Dem Patienten evidenzbasierte Medizin erklären und Stellung nehmen können zu nicht evidenzbasierten Therapieformen (z.B. Vitaminsupplementation, Original Himalaya-Salz, Knoblauch etc.)	K	3	10	73	1,79	0,71					zukunftsrelevant
102	Grenzbereich Soft Skills	Betreuung von Patienten (Aufnahme, Diagnostikeinleitung, Diagnose, Therapie, Entlassung) durchführen	K	3	10	73	1,42	0,62					zukunftsrelevant
103	Grenzbereich Soft Skills	Dokumentation von Befunden und Patientenkontakten vornehmen (ggf. Nutzung von Dokumentationssystemen)	K	3	10	73	1,70	0,68					zukunftsrelevant
104	Grenzbereich Soft Skills	Schreiben und Diktieren von Epikrisen/Arztbriefen	K	3	10	73	2,00	0,71					zukunftsrelevant
105	Grenzbereich Soft Skills	Durchführen einer Kurven-/Patientenvisiten	K	3	10	73	1,88	0,78					zukunftsrelevant

*Konsensusstatement "Praktische Fertigkeiten im Medizinstudium" [...]. Schnabel KP et al, GMS Z Med Ausbild, 2011

Nummer Konsensusstatement*	Organsystem	Fertigkeit	Kern-/Wahllernziel	Tiefendimension WB-Reife	Runde 1: Expertengruppe	n	Mittelwert	Standardabweichung	Runde 2: Expertengruppe	n	Mittelwert	Standardabweichung	Abschlussbewertung
106	Grenzbereich Soft Skills	Formulierung einer klinischen Fragestellung und Methodik, Ergebnisse und Schlussfolgerungen einer Studie beurteilen können inkl. Durchführung einer Literaturrecherche/Datenbankrecherche/ Wissensrecherche/Leitlinienrecherche (Medline, PubMed, Cochrane Library)	K	3	10	72	1,74	0,62					zukunftsrelevant
107	Grenzbereich Soft Skills	Durchführung einer Patientenvorstellung inkl. Untersuchungsbefunde verbal zutreffend beschreiben (klinische Fallpräsentation)	K	3	10	72	1,43	0,52					zukunftsrelevant
108	Grenzbereich Soft Skills	Eigene Reaktionen im Patientenkontakt wahrnehmen können (Selbstreflexion)	K	3	10	72	1,64	0,63					zukunftsrelevant
109	Grenzbereich Soft Skills	Erkennen ethisch konflikthafter Situationen	K	3	10	72	1,76	0,66					zukunftsrelevant
110	Grenzbereich Soft Skills	Erstellen eines Medikamentenplans	K	3	10	72	1,93	0,64					zukunftsrelevant
111	Grenzbereich Soft Skills	Gemeinsame Entscheidungsfindung im Team	K	3	10	72	1,59	0,66					zukunftsrelevant
112	Grenzbereich Soft Skills	Grundlegende Fähigkeit zur kontinuierlichen Aufrechterhaltung und Gestaltung der Arzt-Patienten-Beziehung erwerben	K	3	10	72	1,94	0,75					zukunftsrelevant
113	Grenzbereich Soft Skills	Hausbesuche verschiedener Anlässe bzw. Visiten mit dem/ der Lehrarzt/ Lehrärztin durchführen und wiedergeben	K	3	10	72	2,33	0,76	11	97	1,78	0,74	zukunftsrelevant
114	Grenzbereich Soft Skills	In der Lage sein, die Elemente der Pflegeplanung und -dokumentation für das ärztliche Handeln zu erkennen	K	3	10	72	2,27	0,67	11	97	1,84	0,63	zukunftsrelevant
115	Grenzbereich Soft Skills	Indikationsstellung und Anordnung technischer Untersuchungen	K	2	10	72	1,75	0,88					zukunftsrelevant
116	Grenzbereich Soft Skills	Kollegiales Verhalten und Fähigkeit zur Teamarbeit (interprofessionelle Kommunikation und Kooperation)	K	3	10	72	1,63	0,70					zukunftsrelevant
117	Grenzbereich Soft Skills	Umgang mit Kindern entsprechend ihrem Entwicklungsstand und ihrer psychosozialen Situation	K	3	10	71	1,93	0,70					zukunftsrelevant

*Konsensusstatement "Praktische Fertigkeiten im Medizinstudium" [...]. Schnabel KP et al, GMS Z Med Ausbild, 2011

Nummer Konsensusstatement*	Organsystem	Fertigkeit	Kern-/Wahllernziel	Tiefendimension WB-Reife	Runde 1: Expertengruppe	n	Mittelwert	Standardabweichung	Runde 2: Expertengruppe	n	Mittelwert	Standardabweichung	Abschlussbewertung
118	Grenzbereich Soft Skills	Wissenschaftliches Vortragen inkl. Des Umgangs mit Präsentationsprogrammen	K	3	10	71	2,06	0,67	11	96	1,78	0,73	zukunftsrelevant
119	Grenzbereich Soft Skills	Verfassen einer wissenschaftlichen Arbeit	K	3	10	71	2,34	0,61	12	96	2,20	0,86	zukunftsrelevant
120	Harn-/ Geschlechtsorgane	Urinuntersuchung Teststreifen	K	3	2	47	1,94	0,89					zukunftsrelevant
121	Harn-/ Geschlechtsorgane	Urinuntersuchung Urinsediment	K	2	2	47	2,57	0,74	11	97	2,63	1,08	nicht zukunftsrelevant
122	Harn-/ Geschlechtsorgane	Anlage eines Harnwegkatheters bei Mann und Frau	K	3	2	47	1,94	0,89					zukunftsrelevant
123	Harn-/ Geschlechtsorgane	Betreuung während der Geburt (Bishop-Score vor Geburtseinleitung, Abnabeln, Entwicklung des Kindes, vaginaloperative Entbindungen, Leitung der Plazentarperiode, Postnatale Palpation des Fundusstandes, Beurteilung von Plazenta und Nabelschnur)	K	1	2	47	2,17	0,84	11	96	2,16	1,09	zukunftsrelevant
124	Harn-/ Geschlechtsorgane	Erhebung der Sexualanamnese	W	3	2	47	2,17	0,89					zukunftsrelevant
125	Harn-/ Geschlechtsorgane	Nativpräparat vom Vaginalabstrich (Anfertigung und Beurteilung (Lactobacillen, A-minvaginose, Leukozyten), Kolposkopische Einstellung der Portio am Modell im Praktikumsraum, Entnahme eines zytologischen Abstriches und Ausstrich auf einem Objektträger)	W	1	2	47	2,51	0,75	12	96	3,01	0,97	nicht zukunftsrelevant
126	Harn-/ Geschlechtsorgane	Anschlusses eines Patienten an Nierenersatztherapieverfahren (Dialyse, Hämofiltration)	W	1	2	47	2,43	0,74					zukunftsrelevant
127	Harn-/ Geschlechtsorgane	Nierenpunktion	W	1	2	47	2,85	0,81	12	94	3,00	1,02	nicht zukunftsrelevant
128	Harn-/ Geschlechtsorgane	Palpation des Hodens beim Kind	K	2	2	47	2,28	0,80	12	92	2,34	1,23	zukunftsrelevant
129	Harn-/ Geschlechtsorgane	Patientinnen zur Selbstuntersuchung der Brust anleiten können	K	3	2	47	2,04	0,81					zukunftsrelevant

*Konsensusstatement "Praktische Fertigkeiten im Medizinstudium" [...]. Schnabel KP et al, GMS Z Med Ausbild, 2011

Nummer Konsensusstatement*	Organsystem	Fertigkeit	Kern-/Wahllernziel	Tiefendimension WB-Reife	Runde 1: Expertengruppe	n	Mittelwert	Standardabweichung	Runde 2: Expertengruppe	n	Mittelwert	Standardabweichung	Abschlussbewertung
130	Harn-/ Geschlechtsorgane	Postpartale Versorgung: Betreuung und Überwachung von Mutter und Kind, Wundinspektion nach Episiotomie und Sektio, Beurteilung der laktierenden Mammae und des Laktationsprozesses, Palpation des puerperalen Uterus-Höhenstandes, Anleitung zur Hygiene	W	2	2	47	2,38	0,71	12	92	2,34	0,80	zukunftsrelevant
131	Harn-/ Geschlechtsorgane	Durchführung einer rektalen Untersuchung (Tumor, Douglasschmerz, Stuhlfarbe und -konsistenz) und einen "schonenden" Untersuchungsgang beschreiben können	K	3	2	46	1,63	0,64					zukunftsrelevant
132	Harn-/ Geschlechtsorgane	Anlage und Bewertung des Kardiotokogramms	K	1	2	46	1,43	0,54					zukunftsrelevant
133	Harn-/ Geschlechtsorgane	Überprüfung des errechneten Geburtstermins durch komplementäre Methoden (z.B. Frühultraschallbefunde)	W	1	2	46	2,50	0,84	12	92	2,67	1,00	nicht zukunftsrelevant
134	Harn-/ Geschlechtsorgane	Durchführung und Beurteilung einer Uroflowmetrie	W	1	2	46	2,82	0,71	12	93	3,08	0,96	nicht zukunftsrelevant
135	Harn-/ Geschlechtsorgane	Durchführung und Beurteilung einer Diaphanoskopie des Hodens	W	1	2	46	2,57	0,81	12	93	2,58	1,08	nicht zukunftsrelevant
136	Harn-/ Geschlechtsorgane	Sonographische Restharnbestimmung	W	1	2	46	2,77	0,73	12	94	2,80	1,16	nicht zukunftsrelevant
137	Harn-/ Geschlechtsorgane	Aufarbeitung und Beurteilung eines Spermiogramms	W	1	2	46	2,93	0,83	12	96	3,44	0,98	nicht zukunftsrelevant
138	Harn-/ Geschlechtsorgane	Strukturen der Nieren- und Harnwege im Ultraschall aufsuchen und benennen können	W	3	2	46	2,04	0,73					zukunftsrelevant
139	Harn-/ Geschlechtsorgane	Suprapubischer Blasenkatheter	W	1	2	46	2,26	0,71					zukunftsrelevant
140	Harn-/ Geschlechtsorgane	Untersuchung der männlichen Genitalien	K	3	2	46	2,09	0,86					zukunftsrelevant
141	Harn-/ Geschlechtsorgane	Untersuchung der weiblichen Brust und ihrer Lymphknotenstationen inkl. Inspektion, Palpation und Befundbeschreibung durchführen	K	3	2	46	1,96	0,82					zukunftsrelevant
142	Harn-/ Geschlechtsorgane	Untersuchung der weiblichen Genitalien einschließlich Spekulum (in vitro oder in vivo)	K	1	2	46	2,35	0,71					zukunftsrelevant
143	Haut	Beschreibung von Hauteffloreszenzen	K	3	3	64	1,89	0,57					zukunftsrelevant

*Konsensusstatement "Praktische Fertigkeiten im Medizinstudium" [...]. Schnabel KP et al, GMS Z Med Ausbild, 2011

Nummer Konsensusstatement*	Organsystem	Fertigkeit	Kern-/Wahllernziel	Tiefendimension WB-Reife	Runde 1: Expertengruppe	n	Mittelwert	Standardabweichung	Runde 2: Expertengruppe	n	Mittelwert	Standardabweichung	Abschlussbewertung
144	Haut	Beurteilung von Exanthemen	K	3	3	64	2,02	0,65					zukunftsrelevant
145	Haut	Vorgehen einer Hautbiopsie beschreiben und am Modell durchführen können	W	2	3	64	2,78	0,86	11	101	2,78	0,89	nicht zukunftsrelevant
146	Haut	Dermatologische Ganzkörperuntersuchung durchführen (vollständige Inspektion: Haut behaart/unbehaart, Schleimhäute, Adnexe, auch in Bezug auf Ikterus, Dehydratation, OP-Narben und Venenzeichnung)	K	3	3	64	1,89	0,82					zukunftsrelevant
147	Haut	Dermatologische Hilfsmittel sinnvoll nutzen (Dermatoskop, Glasspatel, Palpation, Holzspatel, Lupe, Sonde) und dermatologischen Befund erstellen können	K	2	3	64	2,02	0,83					zukunftsrelevant
148	Haut	Erkennen von malignen Hauttumoren (Melanom, Basaliom, Spinaliom)	K	1	3	64	1,38	0,49					zukunftsrelevant
149	Haut	Hautmaterial (Schuppen, Sekret) gewinnen und als Präparat anfertigen und anfärben können	W	2	3	64	2,92	0,72					nicht zukunftsrelevant
150	Haut	Inspektion von Nägeln	K	3	3	64	2,15	0,71	11	97	2,48	0,94	zukunftsrelevant
151	Haut	Salbenverbandanlage	K	3	3	64	2,22	0,92					zukunftsrelevant
152	Haut	Spezielle Verfahren der Dermatologie (Atopie-Patch-Test, Wood-Licht, Gewebeprobe, Planimetrie, UV-A-Lampe, Nativpräparat [Pilznachweis], Milben-Dermastoskopie, Diglyoximtest, Trichogramm)	W	1	3	64	2,70	0,71	11	97	3,11	0,83	nicht zukunftsrelevant
153	Haut	Prick- und Epikutantest, Tuberkulin-Test	K	1	3	64	2,03	0,69					zukunftsrelevant
154	Haut	Therapieverfahren in der Dermatologie (Photo- und Photodynamische Therapie, Lasertherapie [ablativ/nicht-ablativ], IPL-Therapie; Dermatochirurgie, lokale Injektions- und Inflitrationstherapien, spezifische Immuntherapie)	W	1	3	64	2,55	0,80	11	99	3,00	0,85	nicht zukunftsrelevant
155	Haut	Untersuchung der Haut und Hautanhangsgebilde	K	3	3	64	1,84	0,62					zukunftsrelevant
156	Herz-Kreislauf	12-Kanal-EKG ableiten (Elektrodenposition, Kabelkonnektion, Patienteninstruktion)	K	3	3	63	1,59	0,71					zukunftsrelevant
157	Herz-Kreislauf	Arteriellen Zugang für invasive Blutdruckmessung anlegen können	W	2	3	63	2,57	0,89	12	94	2,44	0,93	zukunftsrelevant
158	Herz-Kreislauf	Umgang mit arteriellen Zugängen	K	3	3	63	2,37	0,90	12	93	2,15	1,04	zukunftsrelevant

*Konsensusstatement "Praktische Fertigkeiten im Medizinstudium" [...]. Schnabel KP et al, GMS Z Med Ausbild, 2011

Nummer Konsensusstatement*	Organsystem	Fertigkeit	Kern-/Wahllernziel	Tiefendimension WB-Reife	Runde 1: Expertengruppe	n	Mittelwert	Standardabweichung	Runde 2: Expertengruppe	n	Mittelwert	Standardabweichung	Abschlussbewertung
159	Herz-Kreislauf	Anlage venöser Zugang	K	3	3	63	1,33	0,57					zukunftsrelevant
160	Herz-Kreislauf	Anlage zentral-venöser Katheter	K	2	3	63	2,44	0,91	12	92	2,29	0,96	zukunftsrelevant
161	Herz-Kreislauf	Umgang mit ZVK	**Lernziel ist nicht bewertet worden**										-
162	Herz-Kreislauf	Arterielle Punktion und Interpretation der BGA	K	3	3	63	1,92	0,73					zukunftsrelevant
163	Herz-Kreislauf	Auskultation des Herzens	K	3	3	63	1,24	0,53					zukunftsrelevant
164	Herz-Kreislauf	Durchführung eines Belastungs-EKGs	K	2	3	63	2,44	0,76	12	92	2,00	0,80	zukunftsrelevant
165	Herz-Kreislauf	Blutdruckmessung (Seitenvergleich)	K	3	3	63	1,21	0,45					zukunftsrelevant
166	Herz-Kreislauf	Blutkulturentnahme	K	3	3	63	1,43	0,64					zukunftsrelevant
167	Herz-Kreislauf	Blutzuckermessung	K	3	3	63	1,59	0,75					zukunftsrelevant
168	Herz-Kreislauf	Duplexsonographie	W	1	4	53	1,90	0,77					zukunftsrelevant
169	Herz-Kreislauf	Echokardiographie (TTE, TEE)	W	1	4	53	2,02	0,72					zukunftsrelevant
170	Herz-Kreislauf	Infusion richten und verabreichen	K	3	4	53	1,94	0,95	11	101	1,46	0,97	zukunftsrelevant
171	Herz-Kreislauf	Pulsoxymetrie durchführen und beurteilen können	K	3	4	53	1,73	0,81					zukunftsrelevant
172	Herz-Kreislauf	Schellong Test	K	3	4	53	2,85	0,95	11	99	2,68	1,08	nicht zukunftsrelevant
173	Herz-Kreislauf	Seldinger Technik	W	2	4	53	2,19	0,88	11	97	2,33	1,02	zukunftsrelevant
174	Herz-Kreislauf	Umgang mit Portsystem	K	3	4	53	2,04	0,96					zukunftsrelevant
175	Herz-Kreislauf	Inspektion der Jugularvenen (ggf. mit Manöver)	K	3	4	53	2,00	0,76					zukunftsrelevant
176	Herz-Kreislauf	Verschlussdruckmessung	K	2	4	53	2,39	0,83	11	97	2,75	0,98	nicht zukunftsrelevant
177	Herz-Kreislauf	Vollständigen Pulsstatus erheben	K	3	4	53	1,56	0,72					zukunftsrelevant
178	Herz-Kreislauf	Vorbereiten, steriles Aufziehen und die Verdünnung von Medikamenten	K	3	4	53	1,92	0,94					zukunftsrelevant
179	Nervensystem	Orientierende neurologische Untersuchung durchführen können	K	3	3	64	1,28	0,45					zukunftsrelevant
180	Nervensystem	Lumbalpunktion durchführen	K	2	3	64	2,56	0,89	11	96	2,52	1,00	nicht zukunftsrelevant
181	Nervensystem	Neurologische Zusatzuntersuchungen durchführen können (EEG, EMG, Neurographie, evozierte Potenziale, Ultraschall [Duplex, Doppler])	W	1	3	64	2,36	0,68	12	96	2,60	0,79	nicht zukunftsrelevant
182	Nervensystem	Neuroradiologische Untersuchungsmethoden	W	1	3	64	1,95	0,70					zukunftsrelevant

*Konsensusstatement "Praktische Fertigkeiten im Medizinstudium" [...]. Schnabel KP et al, GMS Z Med Ausbild, 2011

Nummer Konsensusstatement*	Organsystem	Fertigkeit	Kern-/Wahllernziel	Tiefendimension WB-Reife	Runde 1: Expertengruppe	n	Mittelwert	Standardabweichung	Runde 2: Expertengruppe	n	Mittelwert	Standardabweichung	Abschlussbewertung
183	Nervensystem	Prüfung der sensiblen, motorischen und vegetativen Qualitäten der Hirnnerven	K	3	3	64	1,42	0,59					zukunftsrelevant
184	Nervensystem	Untersuchung auf meningeale Reizung	K	3	3	64	1,31	0,50					zukunftsrelevant
185	Nervensystem	Untersuchung der Koordination (Ataxieprüfung, Gangproben, Romberg-Versuch, Unterberger-Versuch, Koordination [Ziel- und Feinbewegung, Finger-Nase-Versuch, Knie-Hacken-Versuch, Diadochokinese)	K	3	3	64	1,66	0,67					zukunftsrelevant
186	Nervensystem	Untersuchung der Motorik (Kraftgrade, Muskeleigenreflexe, Fremdreflexe, pathologische Refelxe, Muskeltonus, Rigor, Spastik)	K	3	3	64	1,48	0,53					zukunftsrelevant
187	Nervensystem	Untersuchung der Sensibilität (Haut- und Tiefensensibilität, Zwei-Punkt-Diskrimination, Vibrationsempfinden, Gelenkstellungssinn, Pyramidenbahnzeichen)	K	3	3	64	1,72	0,72					zukunftsrelevant
188	Nervensystem	Untersuchung der Vigilanz, Orientierung und der Sprache	K	3	3	64	1,28	0,45					zukunftsrelevant
189	Organsystem-übergreifende Fertigkeiten	Strukturierte Ganzkörperuntersuchung	K	3	7	51	1,29	0,50					zukunftsrelevant
190	Organsystem-übergreifende Fertigkeiten	Blutentnahme aus peripherer Vene, kapillär (altersabhängig)	K	3	7	51	1,71	0,73					zukunftsrelevant
191	Organsystem-übergreifende Fertigkeiten	Abzessspaltung	W	1	7	51	2,12	0,55					zukunftsrelevant
192	Organsystem-übergreifende Fertigkeiten	Anfertigung einer Wundnaht und Entfernen von Nahtmaterial	K	2	7	51	1,78	0,61					zukunftsrelevant
193	Organsystem-übergreifende Fertigkeiten	Anlage eines Verbandes (z.B. Druckverband und stabilisierenden elastischen Gelenksverband)	K	2	7	51	1,56	0,54					zukunftsrelevant

*Konsensusstatement "Praktische Fertigkeiten im Medizinstudium" [...]. Schnabel KP et al, GMS Z Med Ausbild, 2011

Nummer Konsensusstatement*	Organsystem	Fertigkeit	Kern-/Wahllernziel	Tiefendimension WB-Reife	Runde 1: Expertengruppe	n	Mittelwert	Standardabweichung	Runde 2: Expertengruppe	n	Mittelwert	Standardabweichung	Abschlussbewertung
194	Organsystem-übergreifende Fertigkeiten	Wechseln eines Verbandes unter sterilen Bedingungen (z.B. bei einer sekundär heilenden offenen Bauchwunde)	K	3	7	51	1,73	0,70					zukunftsrelevant
195	Organsystem-übergreifende Fertigkeiten	Anlage einer Vakuumsversiegelung	W	1	7	51	2,65	0,63	12	94	2,62	0,97	nicht zukunftsrelevant
196	Organsystem-übergreifende Fertigkeiten	Applikation von Medikamenten (s.c., i.m., i.a.)	K	3	7	51	1,60	0,60					zukunftsrelevant
197	Organsystem-übergreifende Fertigkeiten	Beurteilung des AZ und EZ	K	3	7	51	1,27	0,45					zukunftsrelevant
198	Organsystem-übergreifende Fertigkeiten	BTM-Anwendung und Verordnung (BTMVV)	K	3	7	51	2,24	0,76	12	93	1,67	0,96	zukunftsrelevant
199	Organsystem-übergreifende Fertigkeiten	Die verschiedene Arten der Infusionstherapie unter Berücksichtigung der Grunderkrankungen unterscheiden, einleiten und durchführen können	K	3	7	51	1,98	0,71	11	101	1,65	0,78	zukunftsrelevant
200	Organsystem-übergreifende Fertigkeiten	Drainage unter Beachtung infektionspräventiver Maßnahmen ziehen können	K	3	7	51	2,20	0,66	12	92	1,69	0,81	zukunftsrelevant
201	Organsystem-übergreifende Fertigkeiten	Wunde beschreiben (Ausdehnung, Alter, Mechanismus, Infektionszeichen) und eine Wunddesinfektion bei septischen und aseptischen Wunden durchführen können	K	3	7	51	1,76	0,55					zukunftsrelevant
202	Organsystem-übergreifende Fertigkeiten	Einleitung und Durchführung einer Narkose	W	1	7	51	2,35	0,52	12	92	1,86	0,78	zukunftsrelevant
203	Organsystem-übergreifende Fertigkeiten	Erkennen kutaner Zeichen von Infektionskrankheiten, Atopie, Allergien, Autoimmunerkrankungen, konsumierender Erkrankungen, internistischer Erkrankungen und sexuell-übertragbarer Erkrankungen	K	3	7	51	1,71	0,54					zukunftsrelevant

*Konsensusstatement "Praktische Fertigkeiten im Medizinstudium" [...]. Schnabel KP et al, GMS Z Med Ausbild, 2011

Nummer Konsensusstatement*	Organsystem	Fertigkeit	Kern-/Wahllernziel	Tiefendimension WB-Reife	Runde 1: Expertengruppe	n	Mittelwert	Standardabweichung	Runde 2: Expertengruppe	n	Mittelwert	Standardabweichung	Abschlussbewertung
204	Organsystem-übergreifende Fertigkeiten	Grundlegende Maßnahmen kennen, die im Rahmen der Dekubitusprophylaxe Anwendung finden und diese ausführen können	K	3	7	51	2,02	0,73					zukunftsrelevant
205	Organsystem-übergreifende Fertigkeiten	Haut- und Schleimhautdesinfektion (Personal und Patient)	K	3	7	51	1,39	0,49					zukunftsrelevant
206	Organsystem-übergreifende Fertigkeiten	Hygienische Händedesinfektion	K	3	7	51	1,16	0,37					zukunftsrelevant
207	Organsystem-übergreifende Fertigkeiten	Chirurgische Händedesinfektion	K	3	7	51	1,33	0,52					zukunftsrelevant
208	Organsystem-übergreifende Fertigkeiten	Hygienisch-mikrobiologische Verfahren zum Erregernachweis in der Umgebung des Patienten und beim Personal durchführen können (Abklatschpräparate usw.)	K	2	7	51	2,14	0,75	12	93	1,95	1,11	zukunftsrelevant
209	Organsystem-übergreifende Fertigkeiten	Infiltration (z.B. subacromial, ISG)	W	1	7	51	2,69	0,62	12	92	2,47	0,91	zukunftsrelevant
210	Organsystem-übergreifende Fertigkeiten	Inspektion des Thorax	K	3	7	51	1,65	0,59					zukunftsrelevant
211	Organsystem-übergreifende Fertigkeiten	Inspektion von Lippen, Zunge, Zahnstatus, Alveolarkamm, Gaumen, Tonsillen, Uvula, Mundhöhlenschleimhaut, Speicheldrüsen und Rachen an einem Probanden vorführen können	K	3	7	51	1,82	0,65					zukunftsrelevant
212	Organsystem-übergreifende Fertigkeiten	Knotentechnik	K	3	7	51	2,20	0,69	12	92	1,71	0,80	zukunftsrelevant
213	Organsystem-übergreifende Fertigkeiten	Leitungsanästhesie	W	1	7	51	2,33	0,52	12	93	1,99	0,81	zukunftsrelevant

*Konsensusstatement "Praktische Fertigkeiten im Medizinstudium" [...]. Schnabel KP et al, GMS Z Med Ausbild, 2011

Nummer Konsensusstatement*	Organsystem	Fertigkeit	Kern-/Wahllernziel	Tiefendimension WB-Reife	Runde 1: Expertengruppe	n	Mittelwert	Standardabweichung	Runde 2: Expertengruppe	n	Mittelwert	Standardabweichung	Abschlussbewertung
214	Organsystem-übergreifende Fertigkeiten	Lokalanästhesie	K	2	7	51	2,02	0,68					zukunftsrelevant
215	Organsystem-übergreifende Fertigkeiten	Nähen	K	3	7	51	2,24	0,71	12	94	1,75	0,81	zukunftsrelevant
216	Organsystem-übergreifende Fertigkeiten	O2-Gabe über Nasensonde	K	3	7	51	1,90	0,83	12	96	1,36	0,73	zukunftsrelevant
217	Organsystem-übergreifende Fertigkeiten	steriles Arbeiten	K	3	7	51	1,39	0,63					zukunftsrelevant
218	Organsystem-übergreifende Fertigkeiten	Umgang mit immunsupprimierten und infektiösen Patienten	K	3	7	51	1,82	0,62					zukunftsrelevant
219	Organsystem-übergreifende Fertigkeiten	Untersuchung auf Hirndruck	K	3	7	51	2,27	0,66	11	96	1,85	0,89	zukunftsrelevant
220	Organsystem-übergreifende Fertigkeiten	Venenstatus erheben	K	3	7	51	2,16	0,61	11	97	1,92	0,81	zukunftsrelevant
221	Organsystem-übergreifende Fertigkeiten	Wunddebridement	K	2	7	51	2,20	0,69	11	97	2,14	0,93	zukunftsrelevant
222	Organsystem-übergreifende Fertigkeiten	Tod eines Patienten sicher feststellen	K	2	7	51	1,86	0,60					zukunftsrelevant
223	Organsystem-übergreifende Fertigkeiten	Ausfüllen einer Todesbescheinigung	K	3	7	51	2,20	0,72	11	99	1,80	0,86	zukunftsrelevant

*Konsensusstatement "Praktische Fertigkeiten im Medizinstudium" [...]. Schnabel KP et al, GMS Z Med Ausbild, 2011

Nummer Konsensusstatement*	Organsystem	Fertigkeit	Kern-/Wahllernziel	Tiefendimension WB-Reife	Runde 1: Expertengruppe	n	Mittelwert	Standardabweichung	Runde 2: Expertengruppe	n	Mittelwert	Standardabweichung	Abschlussbewertung
224	Organsystem-übergreifende Fertigkeiten	Anamnese und körperliche Untersuchung bei interpersoneller Gewalt durchführen und gerichtsfest dokumentieren können	K	3	7	51	2,06	0,73	11	101	2,05	0,95	zukunftsrelevant
225	Psyche	Behandlung Entzugsdelir	K	2	4	53	2,10	0,77					zukunftsrelevant
226	Psyche	Emotionen erkennen und unterscheiden	K	3	4	53	1,60	0,69					zukunftsrelevant
227	Psyche	Umgang mit psychisch Kranken	K	2	4	53	1,80	0,65					zukunftsrelevant
228	Psyche	Zwangseinweisung	K	2	4	53	2,52	0,77	12	96	2,47	1,02	zukunftsrelevant
229	Sinnesorgane	Applikation von Augensalben und -tropfen	K	3	4	53	2,42	0,77	12	94	2,24	1,19	zukunftsrelevant
230	Sinnesorgane	Bindehautabstrich	W	1	4	53	2,67	0,80	12	93	2,74	1,03	nicht zukunftsrelevant
231	Sinnesorgane	Doppeltes Ektropionieren (Desmarres-Haken)	W	1	4	53	2,42	0,79	11	97	2,76	1,07	nicht zukunftsrelevant
232	Sinnesorgane	Durchführung der direkten Ophthalmoskopie	K	2	4	53	2,53	0,68	12	92	2,58	1,01	nicht zukunftsrelevant
233	Sinnesorgane	Hörweitenbestimmung ohne Hilfsmittel (Whispered-Voice-Test)	K	2	4	53	2,55	0,82	11	96	2,72	1,08	nicht zukunftsrelevant
234	Sinnesorgane	Durchführung der indirekten Ophthalmoskopie	K	3	4	53	2,72	0,71	12	92	2,81	0,94	nicht zukunftsrelevant
235	Sinnesorgane	Durchführung der Otoskopie (äußeres Ohr und Trommelfell)	K	3	4	53	1,94	0,79					zukunftsrelevant
236	Sinnesorgane	Durchführung der Rhinoskopie	K	3	4	53	2,47	0,79	12	93	2,73	0,92	nicht zukunftsrelevant
237	Sinnesorgane	Durchführung der Spiegeltechniken des HNO-Fachgebiets (z.B. Laryngoskopie)	W	1	4	53	2,29	0,76					zukunftsrelevant
238	Sinnesorgane	Durchführung der Stimmgabelprüfung nach Weber und Rinne	K	3	4	53	2,28	0,92	12	96	2,52	1,30	nicht zukunftsrelevant
239	Sinnesorgane	Durchführen der Tonschwellenaudiometrie	W	2	5	50	3,26	0,65					nicht zukunftsrelevant
240	Sinnesorgane	Durchführen von Covertests zur Feststellung von latentem und manifestem Schielen	K	3	5	50	3,13	0,59					nicht zukunftsrelevant
241	Sinnesorgane	Einfaches Ektropionieren sowie Fremdkörperentfernung	K	3	5	50	2,15	0,70					zukunftsrelevant

*Konsensusstatement "Praktische Fertigkeiten im Medizinstudium" [...]. Schnabel KP et al, GMS Z Med Ausbild, 2011

Nummer Konsensusstatement*	Organsystem	Fertigkeit	Kern-/Wahllernziel	Tiefendimension WB-Reife	Runde 1: Expertengruppe	n	Mittelwert	Standardabweichung	Runde 2: Expertengruppe	n	Mittelwert	Standardabweichung	Abschlussbewertung
242	Sinnesorgane	Entfernung eines Fremdkörpers aus dem Gehörgang	W	2	5	50	2,19	0,66	11	101	2,60	0,99	nicht zukunftsrelevant
243	Sinnesorgane	Exophthalmometrie nach Hertel	W	1	5	50	2,93	0,65					nicht zukunftsrelevant
244	Sinnesorgane	Fixationsprüfung	K	3	5	50	2,40	0,64	11	99	2,49	1,11	zukunftsrelevant
245	Sinnesorgane	Inspektion des Ohres	K	3	5	50	1,83	0,58					zukunftsrelevant
246	Sinnesorgane	Okulopression	W	1	5	50	2,88	0,54					nicht zukunftsrelevant
247	Sinnesorgane	Orientierende Bestimmung der Sehschärfe (Visustafel)	K	3	5	50	2,32	0,84	11	97	2,42	1,19	zukunftsrelevant
248	Sinnesorgane	Orientierende Perimetrie mittels Finger	K	3	5	50	1,91	0,72					zukunftsrelevant
249	Sinnesorgane	Palpation des Ohres (inkl. Jochbogenansatz, Kiefergelenk und Parotis)	K	3	5	49	2,02	0,56					zukunftsrelevant
250	Sinnesorgane	Prüfung auf Binokularsehen und stereoskopisches Sehen	K	3	5	49	2,41	0,63	11	97	2,90	0,97	nicht zukunftsrelevant
251	Sinnesorgane	Prüfung auf Farbensehen	K	3	5	49	2,63	0,77	11	96	2,84	1,06	nicht zukunftsrelevant
252	Sinnesorgane	Prüfung auf Spontan- und Provokationsnystagmus mit der Frenzelbrille	K	2	5	49	2,35	0,82	12	94	2,74	1,13	nicht zukunftsrelevant
253	Sinnesorgane	Prüfung der Hornhautsensibilität	K	3	5	49	2,57	0,70	12	92	2,59	1,13	nicht zukunftsrelevant
254	Sinnesorgane	Prüfung der Pupillenreaktionen (direkte/indirekte Pupillenreaktion, Konvergenz- und Akkomodationsreaktion)	K	3	5	49	1,53	0,73					zukunftsrelevant
255	Sinnesorgane	Prüfung des Geruchssinns	K	2	5	49	2,89	0,68					nicht zukunftsrelevant
256	Sinnesorgane	Prüfung des Geschmacksinns	K	2	5	49	2,98	0,66					nicht zukunftsrelevant
257	Sinnesorgane	Prüfung des Kontaktlinsensitzes	W	1	5	49	2,83	0,80	12	92	2,78	1,02	nicht zukunftsrelevant
258	Sinnesorgane	Schirmer Test	W	1	5	49	2,80	0,72	12	92	2,90	0,99	nicht zukunftsrelevant

*Konsensusstatement "Praktische Fertigkeiten im Medizinstudium" [...]. Schnabel KP et al, GMS Z Med Ausbild, 2011

Nummer Konsensusstatement*	Organsystem	Fertigkeit	Kern-/Wahllernziel	Tiefendimension WB-Reife	Runde 1: Expertengruppe	n	Mittelwert	Standardabweichung	Runde 2: Expertengruppe	n	Mittelwert	Standardabweichung	Abschlussbewertung
259	Sinnesorgane	Skiaskopie	W	1	6	57	2,96	0,68					nicht zukunftsrelevant
260	Sinnesorgane	Spülen der Tränenwege	W	1	6	57	2,96	0,73	11	101	2,24	0,98	zukunftsrelevant
261	Sinnesorgane	Spülen des Auges	K	3	6	57	2,62	0,89	11	99	2,51	1,05	nicht zukunftsrelevant
262	Sinnesorgane	Tamponieren der Nase	K	2	6	57	2,14	0,66					zukunftsrelevant
263	Sinnesorgane	Thermische Prüfung der Gleichgewichtsorgane	W	2	6	57	2,85	0,64					nicht zukunftsrelevant
264	Sinnesorgane	Untersuchung der Lider, Konjunktiven, Kornea und Skleren mit der Visitenleuchte	K	3	6	57	2,08	0,73	12	92	2,19	1,07	zukunftsrelevant
265	Sinnesorgane	Untersuchung des Kopfes (Inspektion, Palpation, Perkussion)	K	3	6	57	1,30	0,48					zukunftsrelevant
266	Sinnesorgane	Untersuchung des Tränenapparats	K	3	6	57	3,11	0,62					nicht zukunftsrelevant
267	Sinnesorgane	Untersuchung mit der Spaltlampe	K	2	6	57	2,55	0,86	11	97	2,90	0,92	nicht zukunftsrelevant
268	Sinnesorgane	Verband des Auges	K	3	6	57	2,43	0,77	11	97	2,57	0,95	nicht zukunftsrelevant
269	Wachstum, Altern	Anwendung einfacher Testverfahren (geriatrisches Basisassessment, Sturzassessment)	W	2	6	54	1,83	0,61					zukunftsrelevant
270	Wachstum, Altern	Bestimmung des Knochenalters	K	1	6	54	2,96	0,70					nicht zukunftsrelevant
271	Wachstum, Altern	Demonstration und Anleitung der Anwendung von Medikamenten	K	2	6	54	1,37	0,62					zukunftsrelevant
272	Wachstum, Altern	Motorische und psychosoziale Entwicklung von Kindern einschätzen können (Meilensteine: Lächeln, Gehen, Greifen, zum Mund führen, Krabbeln, Umdrehen den verschieden Altersstufen zuordnen können und die Variabilität der Zeitpunkte abschätzen können)	K	2	6	54	2,09	0,73					zukunftsrelevant
273	Wachstum, Altern	Durchführung einer Leichenschau	K	2	6	54	1,41	0,63					zukunftsrelevant

*Konsensusstatement "Praktische Fertigkeiten im Medizinstudium" [...]. Schnabel KP et al, GMS Z Med Ausbild, 2011

Nummer Konsensusstatement*	Organsystem	Fertigkeit	Kern-/Wahllernziel	Tiefendimension WB-Reife	Runde 1: Expertengruppe	n	Mittelwert	Standardabweichung	Runde 2: Expertengruppe	n	Mittelwert	Standardabweichung	Abschlussbewertung
274	Wachstum, Altern	Krankenbett nach den Bedürfnissen/ Wünschen bzw. den Erfordernissen bestimmter Grunderkrankungen einstellen	K	3	6	54	2,26	0,99	12	96	2,15	1,14	zukunftsrelevant
275	Wachstum, Altern	Einen orientierenden psychopathologischen Befund des älteren Menschen erheben können	K	2	6	54	1,89	0,72					zukunftsrelevant
276	Wachstum, Altern	Erhebung einer gezielten Anamnese zu psychiatrischen Symptomen, Depression, Demenz-Symptomen	K	2	6	54	1,80	0,66					zukunftsrelevant
277	Wachstum, Altern	Erstellen und Interpretieren von Perzentilenkurven	K	3	6	54	2,31	0,84	12	93	2,05	0,91	zukunftsrelevant
278	Wachstum, Altern	Exantheme und Enantheme bei Masern, Röteln, Exanthema subitum, Windpocken und Scharlach unterscheiden	K	2	6	54	1,83	0,67					zukunftsrelevant
279	Wachstum, Altern	Grundlagen der Skelettalterbestimmung am Röntgenbild erkennen	W	1	6	54	2,89	0,77					nicht zukunftsrelevant
280	Wachstum, Altern	Impftechnik	K	3	6	54	1,74	0,78					zukunftsrelevant
281	Wachstum, Altern	Anordnung/ Kontrolle der Patientenmobilisation bzw. -lagerung	K	2	6	54	1,93	0,82					zukunftsrelevant
282	Wachstum, Altern	Pediatric Basic Life Support	K	2	6	54	1,70	0,77					zukunftsrelevant
283	Wachstum, Altern	Physikalische Maßnahmen der Fiebersenkung anwenden können	K	3	6	54	2,11	0,88	11	96	1,77	0,95	zukunftsrelevant
284	Wachstum, Altern	Säugling wickeln	K	3	6	54	3,04	0,82					nicht zukunftsrelevant
285	Wachstum, Altern	Spezielle Untersuchungen in der Kinderheilkunde (Neugeborenenscreening, Screeningtest Visus, Screeningtest Entwicklung, Wachstumshormontestung)	W	1	6	54	2,04	0,73					zukunftsrelevant
286	Wachstum, Altern	Physiologische und krankhafte Alternsprozesse anhand komplexer Kasuistik erkennen, die Abgrenzung begründen sowie die Therapieplanung beurteilen und Prognose abschätzen können	K	1	6	54	1,80	0,76					zukunftsrelevant
287	Wachstum, Altern	Geriatrisches Assessment eines Patienten durchführen und ärztliche Versorgung interprofessionell planen	K	2	6	54	1,63	0,62					zukunftsrelevant
288	Wachstum, Altern	Beurteilung des schwangeren Abdomens	W	1	6	54	1,81	0,62					zukunftsrelevant
289	Wachstum, Altern	Pubertätsentwicklung beurteilen können	K	1	6	54	1,96	0,67					zukunftsrelevant

*Konsensusstatement "Praktische Fertigkeiten im Medizinstudium" [...]. Schnabel KP et al, GMS Z Med Ausbild, 2011
